# Increased methylation of the MOR gene proximal promoter in primary sensory neurons plays a crucial role in the decreased analgesic effect of opioids in neuropathic pain

**DOI:** 10.1186/1744-8069-10-51

**Published:** 2014-08-13

**Authors:** Xue-Long Zhou, Li-Na Yu, Yin Wang, Li-Hui Tang, Yu-Nan Peng, Jun-Li Cao, Min Yan

**Affiliations:** 1 Department of Anesthesiology, The Second Affiliated Hospital, School of Medicine, Zhejiang University, Hangzhou, China; 2 Jiangsu Province Key Laboratory of Anesthesilogy, Xuzhou Medical College, Xu Zhou, China

**Keywords:** Neuropathic pain, Epigenetics, Opioids

## Abstract

**Background:**

The analgesic potency of opioids is reduced in neuropathic pain. However, the molecular mechanism is not well understood.

**Results:**

The present study demonstrated that increased methylation of the Mu opioid receptor (MOR) gene proximal promoter (PP) in dorsal root ganglion (DRG) plays a crucial role in the decreased morphine analgesia. Subcutaneous (s.c.), intrathecal (i.t.) and intraplantar (i.pl.), not intracerebroventricular (i.c.v.) injection of morphine, the potency of morphine analgesia was significantly reduced in nerve-injured mice compared with control sham-operated mice. After peripheral nerve injury, we observed a decreased expression of MOR protein and mRNA, accompanied by an increased methylation status of MOR gene PP, in DRG. However, peripheral nerve injury could not induce a decreased expression of MOR mRNA in the spinal cord. Treatment with 5-aza-2′-deoxycytidine (5-aza-dC), inhibited the increased methylation of MOR gene PP and prevented the decreased expression of MOR in DRG, thereby improved systemic, spinal and periphery morphine analgesia.

**Conclusions:**

Altogether, our results demonstrate that increased methylation of the MOR gene PP in DRG is required for the decreased morphine analgesia in neuropathic pain.

## Introduction

The treatment of neuropathic pain (NP) continues to be a major clinical challenge. It is estimated that approximately half of all patients with neuropathic pain fail to receive any relief from their symptoms despite taking prescribed analgesics [1]. Several randomized controlled trials have shown that opioid analgesics provide greater pain relief than that of placebos [2] and comparable pain relief to that of gabapentin and tricyclic antidepressants [2-4]. However, treating neuropathic pain with opioids is often discouraged because of its lower efficacy and the unwanted side effects (such as tolerance and addiction) that can result from long-term and high-dose opioid therapy [5-14]. It is therefore important to understand why opioid analgesia is decreased during neuropathic pain; the answer to this question may provide critical clues for elucidating the mechanisms of neuropathic pain.

Opioids exert their pharmacological and physiological effects by binding to their endogenous receptors, which are found in the central and peripheral nervous systems. Three types of opioid receptors have been cloned: mu (μ), delta (δ), and kappa (κ), and all three of these receptors belong to the G protein-coupled receptor superfamily. Upon agonist binding, these receptors couple to G proteins and affect several signal transduction pathways that are thought to mediate the broad range of functions and pharmacological effects of endogenous and exogenous opioids [15]. Previous studies have suggested that the mu opioid receptor (MOR) plays a key role in mediating the major clinical effects of analgesics, such as the effects of morphine, as well as the tolerance and physical dependence that develops after prolonged opioid administration [16]. Recently, a growing body of evidence has suggested that MOR expression is downregulated in the spinal cord [17-21] and dorsal root ganglion [18,21-24] after nerve injury. This decreased expression of the MOR may partially explain why opioid analgesia is decreased during neuropathic pain. However, the molecular mechanism driving these dynamic changes in MOR expression after nerve injury has not been carefully studied. Some studies have shown that MOR expression can be controlled by manipulating DNA methylation [25-27]. Furthermore, a recent study reported an increase in global DNA methylation in the spinal cord after nerve damage in neuropathic mice [28]. These studies speculated that decreased expression of the MOR during neuropathic pain may be mediated by increased methylation of the MOR gene promoter. In this study, we examined a chronic constrictive injury (CCI) pain model [29], in rodents to demonstrate that increased methylation of the MOR gene proximal promoter in primary sensory neurons plays a crucial role in decreasing morphine analgesia during neuropathic pain.

## Results

### Morphine analgesia was decreased at the peripheral and spinal levels but not decreased at the supraspinal level after nerve injury in neuropathic mice

**Figure 1:**
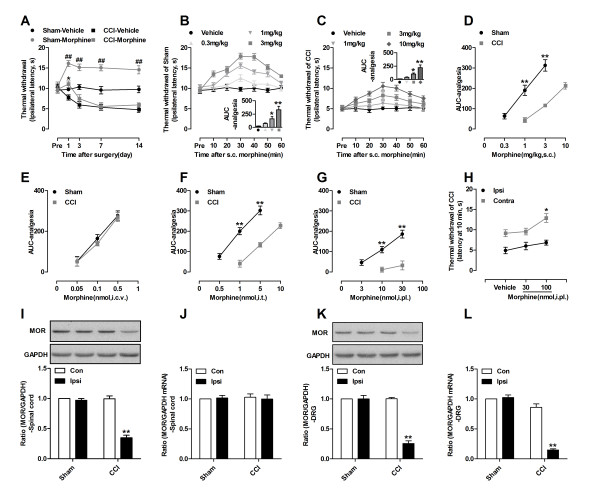
**Systemic, spinal and periphery morphine analgesia decreased in neuropathic mice. (A)** Effects of 1 mg/kg s.c. morphine on TWL in sham-operated and neuropathic pain mice on days -1, 1, 3, 7 and 14 after surgery. Results are presented as TWL at 30 min after s.c. injection of morphine or vehicle. ^##^*p*<0.05 compared with Sham-Vehicle; *p<0.05 compared with CCI-Vehicle, n=8 in each group. **(B, C)** Time course of the effects of s.c. morphine in sham-operated and nerve-injured mice on day 7 after surgery. Results are presented as TWL in seconds, comparison of morphine analgesia by area under the curve (AUC). *p <0.05, **p <0.01 compared with Vehicle, n=8 in each group. **(D-G)** Dose–response curves of s.c. **(D)**, i.c.v. **(E)**, i.t. **(F)** and i.pl. **(G)** morphine in sham-operated and nerve-injured mice on day 7 following surgery. The data are presented as AUC analgesic. **p <0.01 compared with Sham, n=8 mice in each group. **(H)** Effects of ipsilateral i.pl. morphine on contralateral paw withdrawal latency. Morphine (30 or 100 nmol i.pl.) was injected into the ipsilateral paw, and the PWL was measured in both the ipsi- and contralateral paw at 10 min following injection. **(I, K)** Western blot shows that MOR protein were markedly decreased in the spinal cord **(I)** and DRG **(K)** on day 7 after nerve injury, while no MOR protein decrease was observed in sham-operated mice. **(J, L)** qRT-PCR shows that MOR mRNA was heavily decreased in DRG **(L)** on day 7 day after nerve ligation, while no MOR mRNA decrease was observed in the spinal cord **(J)** and sham-operated mice. The ratio of MOR/GAPDH in contralateral spinal cord or DRG sham mice was set at 1 for quantifications. **p <0.01 compared with corresponding contralateral side, n=4 in each group.

Thirty minutes after a subcutaneous (s.c.) morphine (1 mg/kg) injection, which is when the peak analgesic potency is observed, morphine analgesia measured according to the thermal-withdrawal latency (TWL) was significantly reduced in nerve-injured but not sham-operated mice on days 3, 7 and 14 after injury (Figure 1A). When we measured the TWL using various doses of morphine on day 7 after surgery, we observed that in the control sham-operated mice, s.c. morphine dose-dependently increased the TWL and produced significant analgesic action at doses of 1 mg/kg and 3 mg/kg but not 0.3 mg/kg (Figure 1B). In nerve-injured mice, morphine failed to produce a significant analgesic effect at a 1 mg/kg dose. Morphine produced considerable analgesia in nerve-injured mice only at the higher doses of 3 mg/kg and 10 mg/kg (Figure 1C). When we plotted the area under curve (AUC) of analgesia versus the morphine dose, a clear rightward shift in the dose–response curve of s.c. morphine was observed in nerve-injured mice (Figure 1D). However, systemic morphine (e.g., s.c. morphine) is distributed throughout the body and acts at various locations, including peripheral, spinal, and supraspinal sites. We were unable to determine the sites responsible for the decreased analgesic effect of systemic morphine after nerve injury. Therefore, we designed a set of experiments to locate the possible sites that are changed. We administered morphine through various routes [intracerebroventricular- (i.c.v.), intrathecal- (i.t.), and intraplantar (i.pl.)-injection] in control sham-operated and sciatic nerve-injured mice and measured their thermal paw withdrawal responses. We found that i.t. and i.pl. but not i.c.v. morphine produced a clear rightward shift in the dose–response curve of morphine in nerve-injured mice (Figure 1E-G and Additional file 1: Figure S1). These results indicate that the supraspinal component of morphine analgesia remained intact after nerve injury and that the decreased effectiveness of systemic morphine in nerve-injured mice might be caused by reductions in its potency at the peripheral and spinal levels. Regarding i.pl. morphine injection, ipsilateral injection of 100 nmol of morphine only slightly increased the TWL in the injured paw, whereas it considerably increased the TWL on the contralateral side (Figure 1H). This result suggests that the injection of 100 nmol of i.pl. morphine produced a partially systemic effect.

Previous studies have demonstrated decreased expression of the MOR in the dorsal root ganglion (DRG) [18,21-24] and spinal cord [17-21] after peripheral nerve injury. However, this result is controversial. We therefore assessed the expression of MOR proteins and MOR mRNA in the spinal cord and DRG. We found that decreased morphine analgesia was correlated with CCI-induced downregulation of the MOR in the spinal cord and DRG. Peripheral nerve injury but not sham surgery downregulated MOR protein expression in the ipsilateral spinal cord (Figure 1I) and DRGs (Figure 1K) on day 7 after surgery. However, unlike the decreased expression of MOR mRNA in ipsilateral DRGs after nerve injury (Figure 1L), the expression of MOR mRNA in the ipsilateral spinal cord was not decreased (Figure 1J).

### Decreased MOR expression in the DRG may contribute to decreased MOR expression in the spinal cord after nerve injury

**Figure 2:**
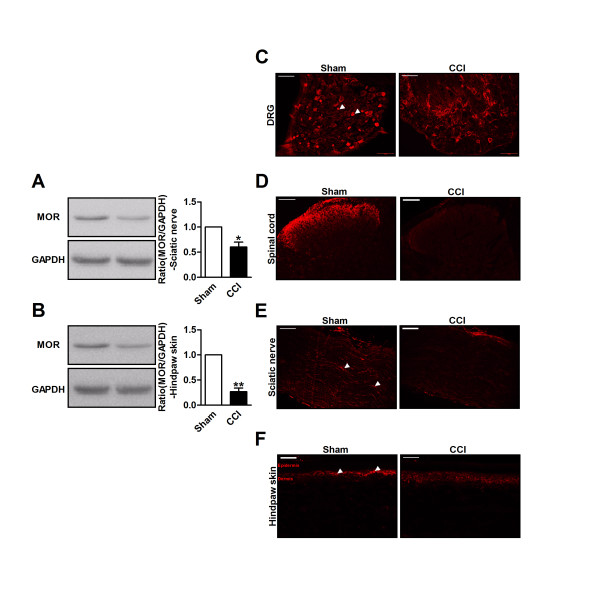
**MOR in primary sensory neuron and its terminals were decreased in neuropathic mice. (A, B)** Western blot shows that MOR protein were markedly decreased in sciatic nerve **(A)** and hindpaw skin **(B)** on day 7 after nerve injury when compared with sham-operated mice. The ratio of MOR/GAPDH in sham mice was set at 1 for quantifications. *p <0.05, **p <0.01, n=4 in each group. **(C-F)** Immunofluorescence staining shows that decreased MOR-labeled neurons in DRG **(C)** and spinal cord dorsal horn **(D)**, decreased MOR-labeled nerve fibers in the sciatic nerve **(E)** and hindpaw skin **(F)** on day 7 after nerve injury. Scales bars= 100 μm.

Peripheral MORs are synthesized in DRG neurons, expressed on the cell bodies of sensory neurons and then transported to central terminals in the superficial dorsal horn and to peripheral terminals of peripheral tissues [12,30]. It has been hypothesized that reduced MOR expression in the dorsal horn of presynaptic afferent terminals results from a drastic decrease in MOR expression in the spinal cord and that this reduced expression contributes to the decreased analgesic potency of i.t. morphine after nerve injury in neuropathic mice. To confirm this hypothesis, we performed western blots and immunofluorescence stains of the MOR in DRG neurons, spinal neurons, sciatic nerve tissue and hindpaw skin on day 7 after nerve injury. Using western blot analysis, we found that MOR expression was significantly decreased at these sites in nerve-injured mice when compared with sham-operated mice (Figures 1I, K and 2A, B). Regarding MOR immunofluorescence staining, there was a substantial decrease in the number of MOR-positive neurons in DRGs on day 7 after nerve injury (Figure 2C). The percentage of MOR-labeled cells in the sham-operated mice was 23.4 ± 5.67; however, the percentage of MOR-labelled cells in the nerve-injured mice was 9.2 ± 2.15 (*p* < 0.01, n = 4). Similarly, MOR expression in the spinal cord was significantly decreased on day 7 after nerve injury, particularly in the superficial lamina (Figure 2D). Many fibers were positively labeled in the sham-operated sciatic nerve tissue and hindpaw skin; however, positively labeled nerve fibers were barely detected after nerve injury (Figure 2E, F).

### Treatment with 5-aza-dC improved the analgesic potency of peripheral, spinal, and systemic morphine in neuropathic mice

**Figure 3:**
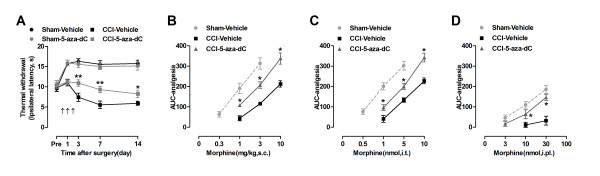
**5-aza-dC improved morphine analgesia in neuropathic mice. (A)** i.t. 5-aza-dC (5 μg, daily for 3 consecutive days, starting 30 minutes before surgery) improved the effects of s.c. 1 mg/kg morphine in nerve-injured mice on days 3, 7 and 14 after surgery. Results are presented as TWL at 30 min after s.c. injection of morphin. *p <0.05, **p <0.05 compared with CCI-Vehicle, n=8 mice in each group. **(B-D)** Dose–response curves of s.c. **(B)**, i.t. **(C)** and i.pl. **(D)** morphine in nerve-injured and 5-aza-dC treatmented mice on day 7 after nerve injury. The data are presented as AUC analgesic. *p <0.05 compared with CCI-Vehicle, n=8 mice in each group.

Peripheral nerve injury has been shown to lead to a decreased expression of the MOR in DRGs [18,21-24]; our experiments confirmed this finding. However, little is known about the regulatory mechanisms of MOR expression in the DRGs after nerve injury. Recently, growing evidence has supported the notion that MOR expression can be controlled by transcriptional regulation mechanisms that involve DNA methylation [25-27]. To investigate whether expression of the MOR gene is mediated by DNA methylation, we treated nerve-injured mice with an inhibitor of DNA methylation, 5′-aza-2′-deoxycytidine (5-aza-dC). After consecutive intrathecal injections of 5-aza-dC (5 μg daily for 3 consecutive days, beginning 30 minutes before surgery), we found that the reduced systemic (Figure 3A) morphine analgesia (1 mg/kg, s.c.) previously observed on days 3, 7 and 14 after nerve injury had improved. When we plotted the AUC of analgesia versus morphine dose on day 7 after 5-aza-dC treatment, the dose–response curves for s.c., i.t. and i.pl. morphine were clearly shifted leftward in the mice treated with 5-aza-dC compared with non-treated mice (Figure 3B-D and Additional file 2: Figure S2).

### Methylation status of the MOR gene promoter after nerve injury

**Figure 4:**
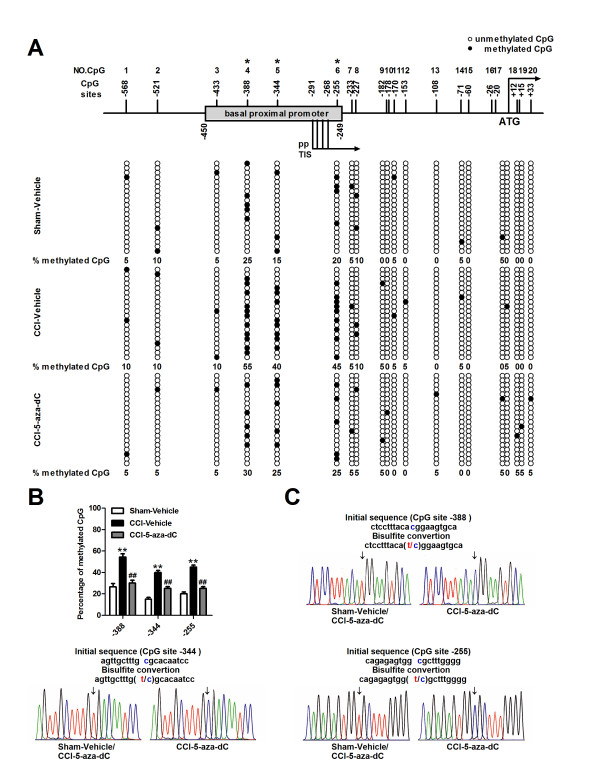
**Methylation statuses of MOR gene proximal promoter (PP) in DRG. (A)** The PP region of the MOR gene contains 20 putative methyl CpG sites from -569 to +33 (with the ATG start codon designated +1). The numbers at the top of the figure (No. CpG) are arbitrary designations to indicate each methyl CpG site. ppTIS indicates the TISs of the major MOR PP containing four sites. Each row of circles represents a single cloned allele, and each circle indicates a single CpG site at a specific location. The methylation statuses of 20 individual clones were analyzed for each mouse. The filled and open circles represent the methylated and unmethylated CpG sites, respectively. **(B)** Methylation changes within the PP region of MOR gene. The percentages of methylation at -388, -344 and -255 CpG sites significantly increased in DRG after nerve injury and reduced after 5-aza-dC treatment (5 μg, daily for 3 consecutive days, starting 30 minutes before surgery). **P<0.01compared with Sham-Vehicle mice; ^##^*p* <0.01 compared with CCI-Vehicle mice, n=4 in each group. **(C)** Samples of the sequence fluorograms obtained using bisulfite genomic sequencing of DNA isolated from the DRG of Sham-Vehicle or CCI-5-aza-dC and CCI-Vehicle mice are shown. Arrows indicate methylated and nonmethylated sequences of CpG site -388, -344, and -255.

**Figure 5:**
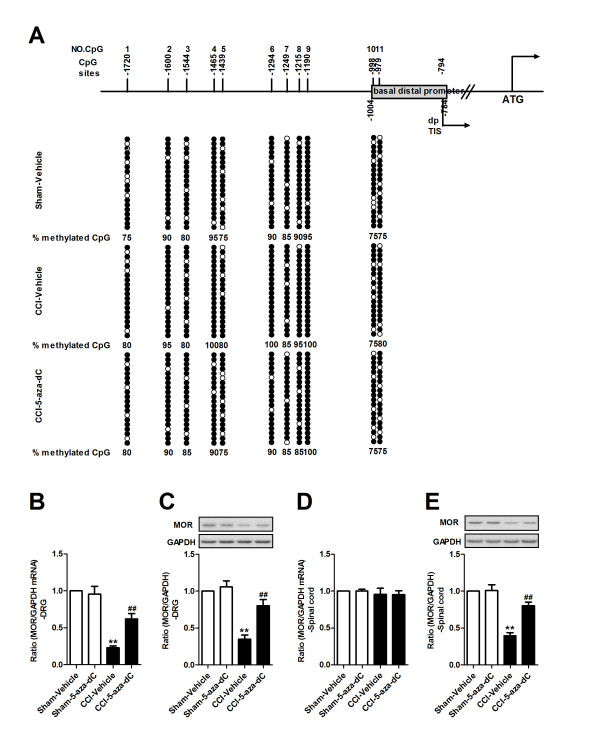
**Methylation statuses of MOR gene distal promoter (DP) in DRG and the expression of MOR change after 5-aza-dC treatment. (A)** The DP region of the MOR gene contains 11 putative methyl CpG sites from -1721 to -784. dpTIS indicates a TIS of the DP. **(B, D)** qRT-PCR show that MOR mRNA was increased in DRG after treatment with 5-aza-dC (5 μg, daily for 3 consecutive days, starting 30 minutes before surgery) **(B)**, while no MOR mRNA increase was observed in the spinal cord **(D)**. The ratio of MOR/GAPDH in Sham-Vehicle mice was set at 1 for quantifications. *p <0.05, **p <0.05 compared with Sham-Vehicle mice; ^##^*p* <0.01 compared with CCI-Vehicle mice, n=4 in each group. **(C, E)** Western blot shows that MOR protein was increased in DRG **(C)** and spinal cord **(E)** after treatment with 5-aza-dC (5 μg, daily for 3 consecutive days, starting 30 minutes before surgery). The ratio of MOR/GAPDH in Sham-Vehicle mice was set at 1 for quantifications. *p <0.05, **p <0.01 compared with Sham-Vehicle mice; ^##^*p* <0.01 compared with CCI-Vehicle mice, n=4 in each group.

As a DNA methylation inhibitor had induced an improvement in the analgesic potency of morphine in neuropathic mice, we attempted to analyze the methylation status of several MOR gene promoters. There are 20 CpG sites in the 5′-flanking region (-569 to +31, which covers the proximal promoter region with the ATG start codon designated as +1) of the MOR gene. The DNA methylation status of multiple MOR gene promoters in the DRG of Sham-Vehicle, CCI-Vehicle, and CCI-5-aza-dC mice were assessed via bisulfite treatment and sequencing analyses on day 7 after nerve injury. The evaluation of 20 individual clones from each mouse revealed that three CpG sites, located at -388, -344 and -255, were highly methylated (over 40% of the clones) after nerve injury and that these three methylated sites were demethylated at least 15% in the presence of 5-aza-dC (Figure 4A, B and Additional file 3: Figure S3A). These three sites are located upstream, close to the proximal promoter (PP)-derived transcription initiation site (TIS). Furthermore, we examined whether the change in methylation status was specific to this PP region, which is known to be a major promoter for the MOR gene, or if it also occurred in other regions. An analysis of the methylation status of the upstream region of the distal promoter (DP) (from -980 to -1721, containing 11 CpG sites) was performed. These sites were highly methylated in the Sham-Vehicle mice (more than 70% methylation, as high as 100%). This hypermethylation was unchanged in the CCI-Vehicle and CCI-5-aza-dC mice (Figure 5A and Additional file 3: Figure S3B). We also found that 5-aza-dC prevented the downregulation of MOR mRNA and MOR proteins in the DRG due to nerve injury (Figure 5B, C). Consistent with the expression pattern of MOR mRNA and MOR proteins in the spinal cord after nerve injury, the expression of MOR mRNA in the spinal cord unchanged (Figure 5D). However, increased expression of MOR protein was observed in the spinal cord after 5-aza-dC treatment in neuropathic mice (Figure 5E). In addition, we analyzed the methylation status of several MOR gene promoters in the spinal cord; no significant changes in methylation status were found in either the proximal or distal promoter of the MOR gene after nerve injury or treatment with 5-aza-dC (Additional file 4: Figure S4).

### Increased MOR expression in the DRG may contribute to the increased MOR expression in the spinal cord induced by 5-aza-dC

**Figure 6:**
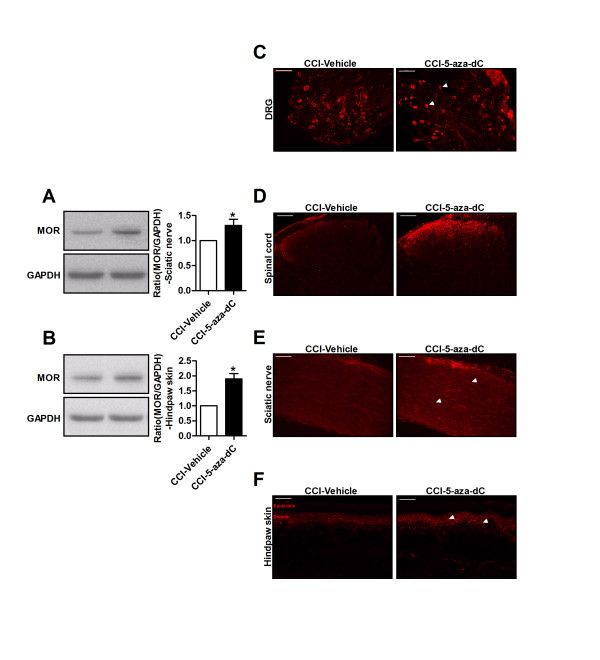
**MOR in peripheral sensory neuron and its terminals were increased after treatment with 5-aza-dC. (A, B)** Western blot shows that MOR protein were markedly increased in sciatic nerve **(A)** and hindpaw skin **(B)** after treatment with 5-aza-dC (5 μg, daily for 3 consecutive days, starting 30 minutes before surgery and detected on day 7 after nerve injury) when compared with CCI-Vehicle mice, *p <0.05, **p <0.01, n=4 in each group. **(C-F)** Immunofluorescence staining shows that increased MOR-labeled neurons in DRG **(C)** and spinal cord dorsal horn **(D)**, increased MOR-labeled nerve fibers in the sciatic nerve **(E)** and hindpaw skin **(F)** after treatment with 5-aza-dC (5 μg, daily for 3 consecutive days, starting 30 minutes before surgery). Scales bars= 100 μm.

Next, we investigated whether the increased expression of MOR proteins after treatment with 5-aza-dC led to an anterograde transport of MOR proteins along the sciatic nerve to peripheral nerve terminals in the skin and/or along the dorsal root to central terminals in the dorsal horn of the spinal cord. We performed western blots and immunofluorescence stains on DRG neurons, spinal neurons, sciatic nerve tissue and hindpaw skin after treatment with 5-aza-dC. We found that MOR expression was increased in mice treated with 5-aza-dC compared with nerve-injured mice according to western blot analysis (Figures 5C, E and 6A, B). On immunofluorescence stains, the number of MOR-positive neurons in the DRG after 5-aza-dC treatment was substantially increased (Figure 6C). The percentage of labeled cells in nerve-injured mice was 8.7 ± 2.24, and the percentage of labelled cells in mice treated with 5-aza-dC was 17.9 ± 4.07 (*p* < 0.01, n = 4). A similar increase in the number of labeled neurons was detected in the spinal cord, sciatic nerve tissue and hindpaw skin of mice treated with 5-aza-dC (Figure 6D-F).

### In vitro, increased methylation of the MOR promoter represses its promoter activity

**Figure 7:**
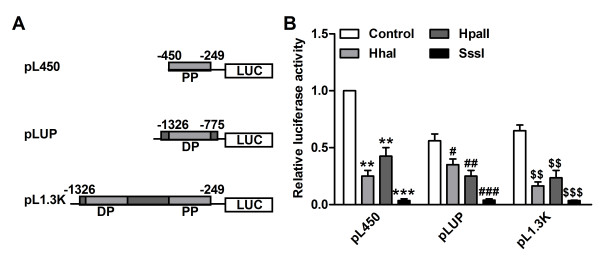
**Repression of MOR promoter-driven transcription by CpG methylation. (A, B)** Three different luciferase (LUC) constructs (pL450, pLup and pL1.3 k) were mock methylated (control) or in vitro methylated with HapI, HpaII (partial) or SssI (full) methylase and transfected into SH-SY5Y cells. The results are given as luciferase activity normalized against cotransfected pCH110 β-galactosidase activity. The data shown are the means of three independent experiments with at least two different plasmid preparations. The control with none methylase was set at 1 for quantifications. ^#^*P*<0.05, ^**’##’$$^*P*<0.01, ^***,###,$$$^*P*<0.001 compared with control, n=4 in each group.

Finally, the relevance of the methylation status of the MOR gene promoter was investigated using reporter gene analyses. To mimic the endogenous methylation status of the MOR gene promoter, MOR promoter plasmids [31] fused with luciferase containing pL450 (PP only, -450 to -249), pLup (DP only, -1,326 to -775) or pL1.3 k (DP and PP, -1,326 to -249) (Figure 7A) were methylated using either partial methylase HpaI, HpaII or full methylase SssI in vitro. Then, the resulting in vitro-methylated MOR promoter/reporter constructs were individually transfected into SH-SY5Y cells. An unmodified promoter (control) was also introduced into SH-SY5Y cells. Partial methylation of the luciferase constructs partially reduced promoter activity compared with the control constructs, whereas full methylation of the constructs completely suppressed promoter activity (Figure 7B). These in vitro methylation data suggest that methylation of the promoter may contribute to the repression of MOR gene transcription in a methylation density-dependent manner.

## Discussion

Neuropathic pain due to peripheral nerve injury is often less responsive to opioids in clinical and experimental animal models. Furthermore, the present study has shown that increased methylation of the MOR gene proximal promoter in primary sensory neurons plays a crucial role in the decreased analgesic effect of opioids in neuropathic mice.

### Opioids for neuropathic pain

In clinical practice, opioids often fail to provide effective analgesia for neuropathic pain [5-8]. Similar results have been observed in experimental animal models [9-14]. Consistent with those findings, the present study observed a marked decrease in the analgesic potency of systemic (s.c.) morphine in mice with sciatic nerve injury-induced neuropathy. Furthermore, our study revealed that the decreased effectiveness of systemic morphine in nerve-injured mice was caused by reduced potency at the peripheral and spinal levels but not at the supraspinal level. This result is consistent with that of a previous study by Rashid et al. [24]. In the present study, we also found that the loss of peripheral morphine analgesia was injury-specific; however, a previous study reported that i.pl. morphine was more effective in the ipsilateral paw than the contralateral paw in rats with spinal nerve ligation-induced neuropathy [11]. It is worth noting that the dose of morphine that produced this effect (200 μg, i.pl.) in the previous study was much higher than the dose used in our study (30 nm, which is approximately 1 μg, i.pl.). The systemic effects of such higher doses cannot be excluded. In our experiment, an ipsilateral injection of 100 nmol of i.pl. morphine produced analgesia in the contralateral paw, indicating the presence of systemic effects at this higher dose (Figure 1H). Nevertheless, other factors, such as the use of different neuropathy models or different species of animals, may be responsible for the discrepancies between our results and those of previous studies.

### The MOR and neuropathic pain

Previous studies have shown that the downregulation of MOR expression that occurs in the spinal cord [17-21] and DRG [18,21-24] may contribute to decreased opioid analgesia after nerve injury. However, the specific sites where decreased MOR expression occurs remain controversial. To determine the specific sites where decreased MOR expression occurs after peripheral nerve injury, we examined MOR expression in the spinal cord and DRG following sciatic nerve injury. Consistent with previous behavioral data, we found that nerve injury drastically decreased MOR expression in the spinal cord and DRG on day 7 after surgery. However, we also found that MOR expression was decreased at both the protein and mRNA levels in the DRG but not the spinal cord. There was no decreased expression of MOR mRNA in the spinal cord after nerve injury. This result is partially consistent with that of a recent study by Lee et al. [23], which reported that MOR expression was decreased at both the protein and mRNA levels in the DRG on day 7, but remained unchanged at the protein level in the spinal cord until day 35 following L5 spinal nerve ligation (SNL). Our finding of a decrease in MOR expression in the spinal cord on day 7 following peripheral nerve injury is consistent with numerous previous reports [17,18,20,32]. For example, Goff et al. [32] reported a drastic decrease in MOR expression in the spinal cord 7 days following tight ligation of the sciatic nerve. In addition, Zhang et al. [18] reported downregulation of the MOR in rats 7 days after peripheral axotomy. However, the report by Zhang et al. involved a considerable species difference with regard to the time of reduced MOR expression following axotomy. Whereas axotomy caused a reduction in spinal cord MOR expression in rats on day 7, axotomy caused a decrease in spinal cord MOR expression in monkeys on day 14 after nerve injury. We speculate that species difference may also accounts for the different time of reduced MOR expression observed in our study of mice compared to the SNL experiments in rats by Lee et al. [23]. Moreover, the use of distinct neuropathy models might also underlie these differences.

In addition, peripheral MORs are synthesized in DRG neurons and expressed on cell bodies of sensory neurons and then transported to central terminals in the superficial dorsal horn and peripheral terminals in peripheral tissues [12,30]. There may be a reduction in MOR expression in the presynaptic afferent terminals of the spinal dorsal horn as a result of the drastic decrease in MOR expression that occurs in the spinal cord; this process may contribute to the decreased analgesic potency of i.t. morphine observed in neuropathic mice. Several lines of evidence support this hypothesis. Besse et al. [33] reported that peripheral nerve injury results in a loss of spinal MORs, which are predominately distributed in the superficial dorsal horn. As previously reported [34-36], MOR expression occurs on both dorsal horn neurons and the presynaptic terminals of laminas I and II (both of which comprise the superficial dorsal horn). Lamina I and the outer portion of lamina II contain a dense plexus of substance P-containing axons, most of which are primary afferent terminals. Moreover, Kondo et al. [37] found that the analgesic effect of opioids in the spinal cord may be mediated by opioid receptors located in C-fiber terminals. On western blotting and immunofluorescence staining of DRG neurons, spinal neurons, sciatic nerve tissue and hindpaw skin on day 7 after nerve injury, we observed decreased expression of MOR proteins in the cell bodies of primary sensory neurons in the DRG after nerve injury. This decreased MOR expression may reduce the transport of morphine to both the peripheral and central terminals of primary sensory neurons in the DRG, decreasing the analgesic effect of spinal and peripheral morphine.

### Methylation modification and the MOR

Epigenetic control, which includes DNA methylation and the histone modifications acetylation and methylation, alters the ability of transcriptional machinery to access DNA, thereby regulating gene expression [38]. Recently, Uchida et al. reported that nerve injury upregulates neuron restrictive silencer factor (NRSF) expression and promotes NRSF binding to the neuron-restrictive silencer element (NRSE) within the MOR gene in DRG neurons. As NRSF binds to NRSE, it represses the transcription of the MOR gene through HDAC-mediated mechanisms [39]. Other than the histone modification mechanisms, the MOR has also been reported to be regulated via DNA methylation in opiate users and this regulation may cause pain [40-42]. However, it is unclear whether DNA methylation is critical for the regulation of MOR expression after nerve injury. In the present study, we showed that increased methylation of the MOR gene proximal promoter in the DRG plays a crucial role in the regulation of MOR expression under neuropathic pain conditions.

In mammals, there are two general mechanisms by which DNA methylation inhibits gene expression [43]. The first mechanism involves modifying cytosine bases to directly inhibit the association of DNA binding factors due to steric hindrance. The second and more important mechanism of inhibition involves various proteins that recognize methylated CpG sites and recruit transcriptional co-repressor molecules to silence transcription. The silencing of methylated promoters usually requires Methyl-CpG-binding proteins (MBPs), which specifically recognize methylated CpG sites. To date, five MBPs have been identified: MBD1, MBD2, MBD3, MBD4, and MeCP2. MeCP2 can bind to a single methylated CpG site, whereas other MBPs [e.g., Methyl-CpG-binding proteins 1 (MeCP1)], generally bind to DNA containing at least 12 symmetrically methylated CpGs [44]. Because only three scattered CpGs (at -388, -344, and -255) out of the 20 CpG sites in the MOR promoter showed changes in their methylation status, we believe that MeCP2 may serve as a possible mediator of transcriptional repression of the MOR gene. Regarding the co-repressor molecules, two recent studies reported that MeCP2 recruits mSin3A and Brg1 to generate a transcriptionally repressed chromatin structure to inhibit expression of the MOR gene [25,45].

## Conclusion

In conclusion, the present study demonstrated that DNA methylation directed epigenetic gene silencing of MOR genes in the DRG is responsible for the decreased efficacy of opioids after nerve injury. Elucidation of regulatory mechanisms for DNA methylation after nerve injury might provide novel therapeutic targets for the neuropathic pain.

## Materials and methods

### Experimental animals

Experiments were performed on adult (weighing 20–25 g) male C57BL/6 J mice. Animals were obtained from Experimental Animal Center of Zhejiang University and were on a 12:12 light–dark cycle with a controlled room temperature (23-24°C, 60-70% relative humidity), and received food and water ad libitum. All experiments were performed in according with the guidelines of the International Association for the Study of Pain [46] and were approved by the Animal Research Committee of Zhejiang University.

### Drug application

Morphine hydrochloride and 5-aza-2′-deoxycytidine (5-aza-dC) were purchased from Sigma (St. Louis, MO) were dissolved in physiological saline. Saline was used for control injections. Intraplantar injections (i.pl.) were given using a Hamilton microsyringe connected to polyethylene tubing with a 30-gauge hypodermic needle at the tip. The volume of injection was 20 μl in thermal paw withdrawal tests. The intrathecal injections (i.t.) were performed freehand between spinal L5 and L6 segments according to the method of Hylden and Wilcox [47]. The exact placement of the drug substances was checked by a quick flicking motion of the mouse’s tail upon entry of needle. The Intracerebroventricular injections (i.c.v.) were carried out into the left lateral ventricle of mice. Injections were performed using a Hamilton microsyringe fitted with a 26-gauge i.c.v. needle according to the method of Haley and McCormick [48]. The site of injection was 2 mm caudal and 2 mm lateral to the bregma and 3 mm in depth from the skull surface. Both i.t. and i.c.v. injections were given in a volume of 5 μl. The mice received the subcutaneous injections (s.c.) in a volume of 0.1 ml/10 g of body weight. All doses of drugs are based on the results of preliminary experiments.

### Chronic constrictive injury model

Chronic constrictive injury (CCI) model was performed following the method of Bennett and Xie. In brief, mice were anesthetized with sodium pentobarbital (40 mg/kg, intraperitoneal injection). Left sciatic nerve was exposed at midthigh level through a small incision, and a unilateral constriction injury just proximal to the trifurcation was performed with three loose ligatures using a 5–0 silk thread (spaced at a 1-mm interval). In sham-operated animals, the nerve was exposed but not ligated. The incision was closed in layers, and the wound was treated with antibiotics.

### Measurement of thermal hyperalgesia

Thermal hyperalgesia was measured using the thermal-withdrawal latency (TWL) according to the method described previously [49]. In brief, mice were placed in clear plastic chambers (7-9-11 cm) and allowed to acclimatize to the environment for 1 h before drug application. The heat source was focused on a portion of the hindpaw, which was flush against the glass, and a radiant thermal stimulus was delivered to that site. The nociceptive endpoints in the radiant heat test were the characteristic lifting or licking of the hind paw. The time to the endpoint was considered the TWL. The radiant heat intensity was adjusted to obtain basal TWL of 10–12 s. An automatic 20 s cutoff was used to prevent tissue damage. After 1 h of adaptation, morphine was injected s.c., i.t., i.c.v., or i.pl., and the thermal withdrawal latencies were measured at every 10-min interval until 60 min. AUC analgesic was measured by deducting the area under the time-response curve of saline (AUC-saline) from the area under the time-response curve of morphine (AUC-morphine). In some experiments, the paw withdrawal latencies at 10 or 30 min after drug administration were measured. The behavioral testing was performed by an investigator blinded to the treatment.

### Real-time quantitative RT-PCR (qRT-PCR)

Under deep anesthesia (sodium pentobarbital, 60 mg/kg, intraperitoneal injection), L4-L5 DRGs and L4-L5 spinal cord segment was quickly removed. Total RNA was isolated with TRIzol reagent (Invitrogen) according to the manufacturer’s instructions. cDNA was then synthesized using a Thermo Scientific Verso cDNA synthesis kit (ABgene, Thermo Scientific) with oligo(dT) primer. Quantitative RT-PCR (qRT-PCR) was performed with a DyNAmo Flash SYBR Green qPCR kit (Thermo Scientific). The thermal cycle conditions used to assess the expression of MOR was: 95°C for 3 minutes; 40 cycles of 95°C for 15 seconds, 58°C for 30 seconds, and 72°C for 30 seconds and finally 30 min at 72°C with the primers: MOR (qRT-PCR), forward TCCTGGTCATGTATGTGATTGT A AGA and reverse CGTGCTAGTGGCTAAGGCATCT; GAPDH, forward TATGACTCCACTCACGGCAAAT and reverse GGGTCTCGCTCCTGGAAGAT. Relative mRNA levels were calculated using the 2 - ΔΔCT method.

### Western blot analysis

Under deep anesthesia, the L4-L5 DRGs and L4-L5 spinal cord segment, sciatic nerves and plantar surface of hindpaw skin of mice were quickly extracted and stored in liquid nitrogen. Tissue samples were homogenized in lysis buffer (12.5 μl/mg tissue) containing a mixture of protease inhibitors (Roche) and PMSF (Sigma). After incubating in ice for 30 min, samples were centrifuged at 10,000 rpm for 15 min at 4°C. The supernatants were used for Western blotting. Equal amount of protein (~30 μg) was loaded and separated in 10% Tris-Tricine SDS-PAGE gel. The resolved proteins were transferred onto polyvinilidene difluoride (PVDF) membranes (Amersham Bioscience). The membranes were blocked in 5% non-fat milk for 1 h at room temperature (RT), and incubated overnight at 4°C with rabbit anti-MOR antibody (Neuromics, 1:1,000) or anti-GAPDH antibody (Cell Signaling Technology, 1:2000) primary antibody. The blots were then incubated with the secondary antibody, goat anti-rabbit IgG conjugated with horseradish peroxidase (HRP) (1:1000, Cell Signaling Technology), for 2 h at room temperature. Signals were finally visualized using enhanced chemiluminescence (ECL, Pierce) and the blots were exposed by The ChemiDoc™ XRS + image system (Bio-Rad; Hercules). All Western blot analysis was performed at least three times, and consistent results were obtained. Western blot densitometry analysis of signal intensity was performed using Quantity One 4.6.2 (Bio-Rad; Hercules) and levels of MOR from densitometry were normalized to GAPDH. The blot density from control groups was set as 100%.

### Immunohistochemistry

Mice were deeply anesthetized and undergone sternotomy, intracardially perfuse with 20 ml saline followed by 4% ice-cold paraformaldehyde in 0.1 mol/l phosphate buffer (PB). L4-L5 DRGs, L4-L5 spinal cord segments, sciatic nerves, and plantar surface of hindpaw skin were removed, post-fixed in 4% paraformaldehyde for 3 h, and subsequently allowed to equilibrate in 30% sucrose in PB overnight at 4°C. DRG and transverse spinal cord sections (15 μm), sciatic nerve sections (10 μm) and skin sections (40 μm) were cut on a cryostat and every fifth section was collected in PB. After washing in phosphate buffer saline (PBS), the tissue sections were incubated in PBS containing 5% normal goat serum and 0.3% TritonX-100 at room temperature for 30 min. The sections were incubated overnight at 4°C with rabbit anti-MOR antibody (Neuromics, 1:500) followed by Rhodamine Red™-X goat anti-rabbit IgG (Invitrogen, 1:500) for 2 h at room temperature. Nonspecific staining was determined by excluding the primary antibodies. Sections were rinsed, mounted, and cover-slipped with glycerol containing 2.5% of anti-fading agent DABCO (Sigma) and stored at -20°C in the dark. Images were captured using a fluorescent microscope (DMIRB, Leica, Germany). The number of immunoreactive neuronal profiles was counted in a blinded fashion. Twenty sections were selected from four mice in each group. For DRG sections, Neuronal cell bodies were identified by the presence of a nucleus. The density threshold for positive staining was determined by averaging three cell bodies in each section that were judged to be minimally positive. All neurons for which the mean density exceeded the threshold were counted as positive, and the positive cells were expressed as a percentage of total counted neurons.

### Methylation analysis

Genomic DNA from the DRG was isolated using the Wizard Genomic DNA Purification Kit (Qiagen) and linearized with the restriction enzyme EcoRV. Bisulfite treatment of DNA was carried out according to the manufacturer’s recommendations (EZ DNA Methylation-Gold Kit; Zymo Research). The resulting bisulfite-modified DNA was amplified by PCR. The PCR conditions were as follows: 94°C for 2 min, followed by 35 cycles of 94°C for 30 seconds, 60°C for 30 seconds, and 72°C for 1 min, and finally 30 min at 72°C. After PCR amplification, the PCR products were purified using a gel extraction kit (QIAGEN) and cloned into the pCR2.1-TOPO vector (Invitrogen) according to the manufacturer’s instructions. Twenty clones containing an insert of the correct size from each mouse were randomly chosen for DNA sequencing. The primers specific for methylated (methylation-specific PCR [MSP]) MOR DNA were designed as follows: MOR (PP), forward GTGGGTAAAGGATAATATTAATAATTTT and reverse CAACTTACAAAAACTAAAAAATCAAAAC; MOR (DP), forward GAGAAGAAGATTGAGGTAAAGTAGTA T and reverse CATACCAAATCTACTCTCCTAAACCTAC.

### In vitro methylation of reporter plasmid and reporter gene assays

In vitro methylation of reporter plasmids was carried out as reported previously [25]. Briefly, methylases SssI, HpaI and HpaII were used to methylate MOR promoter/luciferase reporter constructs following the recommendations of the manufacturer (New England Biolabs). Complete methylation was determined by digesting the DNA constructs with the methylation-sensitive restriction enzyme Bstu1, HpaI, HpaII (New England Biolabs) respectly and running the products on an agarose gel. Only DNA that was completely methylated was used. The construction of all luciferase fusion plasmids (pL450, pLup, and pL1.3 k) used in this study has been described previously [31]. SH-SY5Y cells were plated 24 h prior to transfection at a density of 3×10^5^ cells/well in six-well culture plates. Transfection was carried out using the effectene transfection reagent (QIAGEN) as described by the manufacturer. Cells were washed and lysed with lysis buffer (Promega) 48 h after transfection. To correct differences in transfection efficiency, a one-fifth molar ratio of a pCH110 plasmid (Amersham Biosciences) containing the β-galactosidase gene under the simian virus 40 promoter was included in the transfection to normalize values. The luciferase and galactosidase activities of each lysate were determined as described by the manufacturers (Promega and Tropix, respectively). Each normalized value represents the average of at least three independent determinations.

### Statistical analysis

Data are expressed as mean±SEM. Statistical analysis between two samples was performed using Student *t* test. Statistical comparison of more than two groups was performed using one-way ANOVA followed by a Tukey *post hoc* test. The significance of any differences in thermal latency in behavior test was assessed using two-way ANOVA. “Time” was treated as “within subjects” factor and “treatment” was treated as a “between subjects” factor. The area under the pain threshold change *versus* time curve was calculated by GraphPAD Prism5 (Graph Pad Software, Inc., San Diego, CA) in some behavioral test. Statistical analyses of data were generated using GraphPAD Prism 5. All *p* values given are based on two-tailed tests. A value of *p* less than 0.05 was considered as statistically significant.

## Competing interests

The authors declare that they have no competing interests.

## Authors’ contributions

XLZ, YNP, YW and LHT carried out the experiment and analyzed the data. XLZ and YNP together conceived the study, and participated in its design. LNY coordinated and supervised the experiments. JLC and MY supervised the experiments and wrote the manuscript. All authors have read and approved the final version of the manuscript.

## Supplementary Material

Additional file 1: Figure S1 Spinal and periphery morphine analgesia decreased in neuropathic pain mice. Time course of i.c.v. (A and B), i.t. (D and E) and i.pl. (G and H) morphine in sham-operated and nerve-injured mice at 7 days after surgery. Results are presented as TWL in seconds, comparison of morphine analgesia by AUC. *P<0.05, **P<0.01 compared with Vehicle, n=8 in each group. Dose–response curves of i.c.v. (C), i.t. (F) and i.pl. (I) morphine in sham-operated and nerve-injured mice at 7 days following nerve injury. The data are presented as AUC analgesic. **P<0.01 compared with sham, n=8 in each group. Click here for file

Additional file 2: Figure S2 5-aza-dC improved morphine analgesia in neuropathic pain mice. Time course of i.pl. (A and B), i.t. (D and E) and s.c. (G and H) morphine in nerve-injured and 5-aza-dC (5 μg daily for 3 consecutive days, starting 30 minutes before surgery) treatmented mice on day 7 after surgery. Results are presented as TWL in seconds, comparison of morphine analgesia by AUC. *P<0.05, **P<0.01 compared with Vehicle, n=8 in each group. Dose–response curves of i.pl. (C), i.t.(F and s.c. (I) morphine in nerve-injured and 5-aza-dC treatmented mice on day 7 following nerve injury. The data are presented as AUC analgesic. *P<0.05 compared with CCI-Vehicle, n=8 in each group. Click here for file

Additional file 3: Figure S3 A Methylation statuses of MOR gene PP in DRG. The percentages of methylation at CpG sites in the MOR promoter from the region of base pairs -569 to +33. -388, -344 and -255 CpG sites significantly increased in DRG after nerve injury and 5-aza-dC reduced the hypermethylation status. **P<0.01 compared with Sham-Vehicle mice, ##*P*<0.01compared with CCI-Vehicle mice, n=4 in each group. B, Methylation statuses of MOR gene DP in DRG. The percentages of methylation at CpG sites in the MOR promoter from the region of base pairs -1721 to -780. No CpG site has a significant change. n=4 mice in each group. Click here for file

Additional file 4: Figure S4 A and B, methylation changes within the PP region of spinal MOR gene. No CpG site has a significant change. n=4 in each group. H, methylation changes within the DP region of spinal MOR gene. No CpG site has a significant change. n=4 in each group. Click here for file
