# Supplementary figures and images for: Increased methylation of the MOR gene proximal promoter in primary sensory neurons plays a crucial role in the decreased analgesic effect of opioids in neuropathic pain
Source: Mol Pain. 2014 Aug 13;10:51. doi: 10.1186/1744-8069-10-51 (PMC4137045; doi:10.1186/1744-8069-10-51)

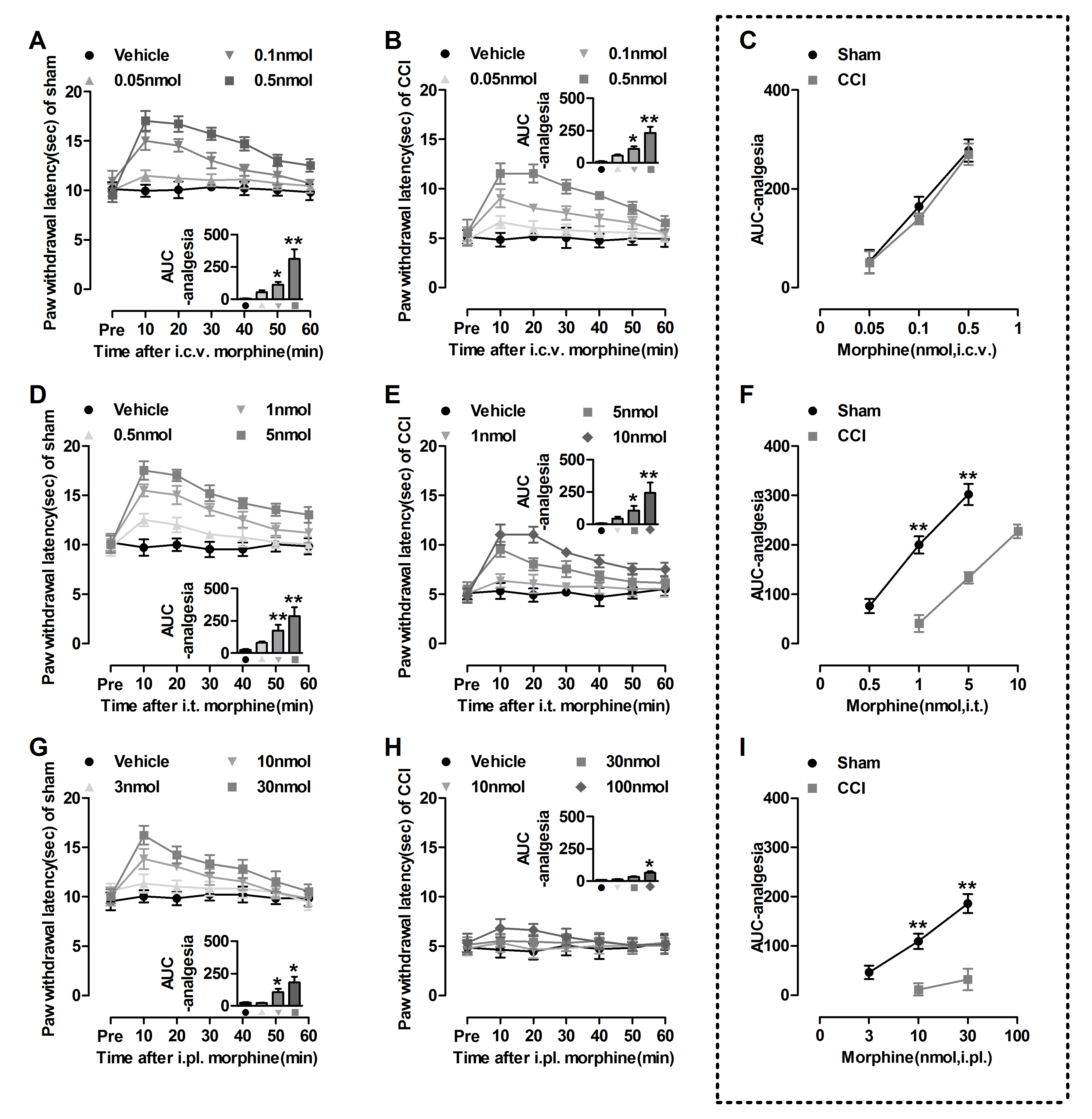

Supplement: Additional file 1: Figure S1 — Spinal and periphery morphine analgesia decreased in neuropathic pain mice. Time course of i.c.v. (A and B), i.t. (D and E) and i.pl. (G and H) morphine in sham-operated and nerve-injured mice at 7 days after surgery. Results are presented as TWL in seconds, comparison of morphine analgesia by AUC. *P<0.05, **P<0.01 compared with Vehicle, n=8 in each group. Dose–response curves of i.c.v. (C), i.t. (F) and i.pl. (I) morphine in sham-operated and nerve-injured mice at 7 days following nerve injury. The data are presented as AUC analgesic. **P<0.01 compared with sham, n=8 in each group. [file 1744-8069-10-51-S1.tiff]

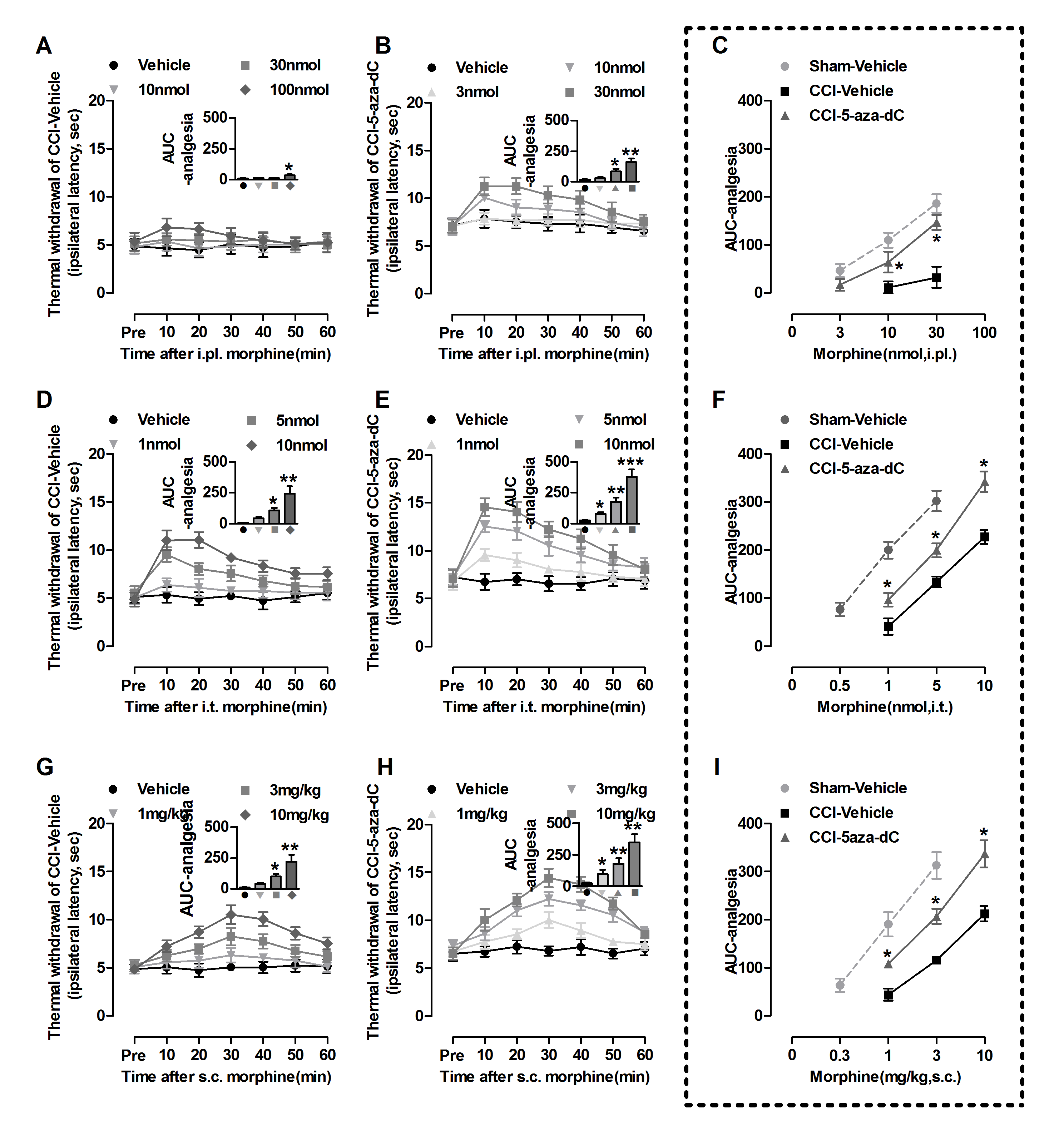

Supplement: Additional file 2: Figure S2 — 5-aza-dC improved morphine analgesia in neuropathic pain mice. Time course of i.pl. (A and B), i.t. (D and E) and s.c. (G and H) morphine in nerve-injured and 5-aza-dC (5 μg daily for 3 consecutive days, starting 30 minutes before surgery) treatmented mice on day 7 after surgery. Results are presented as TWL in seconds, comparison of morphine analgesia by AUC. *P<0.05, **P<0.01 compared with Vehicle, n=8 in each group. Dose–response curves of i.pl. (C), i.t.(F and s.c. (I) morphine in nerve-injured and 5-aza-dC treatmented mice on day 7 following nerve injury. The data are presented as AUC analgesic. *P<0.05 compared with CCI-Vehicle, n=8 in each group. [file 1744-8069-10-51-S2.tiff]

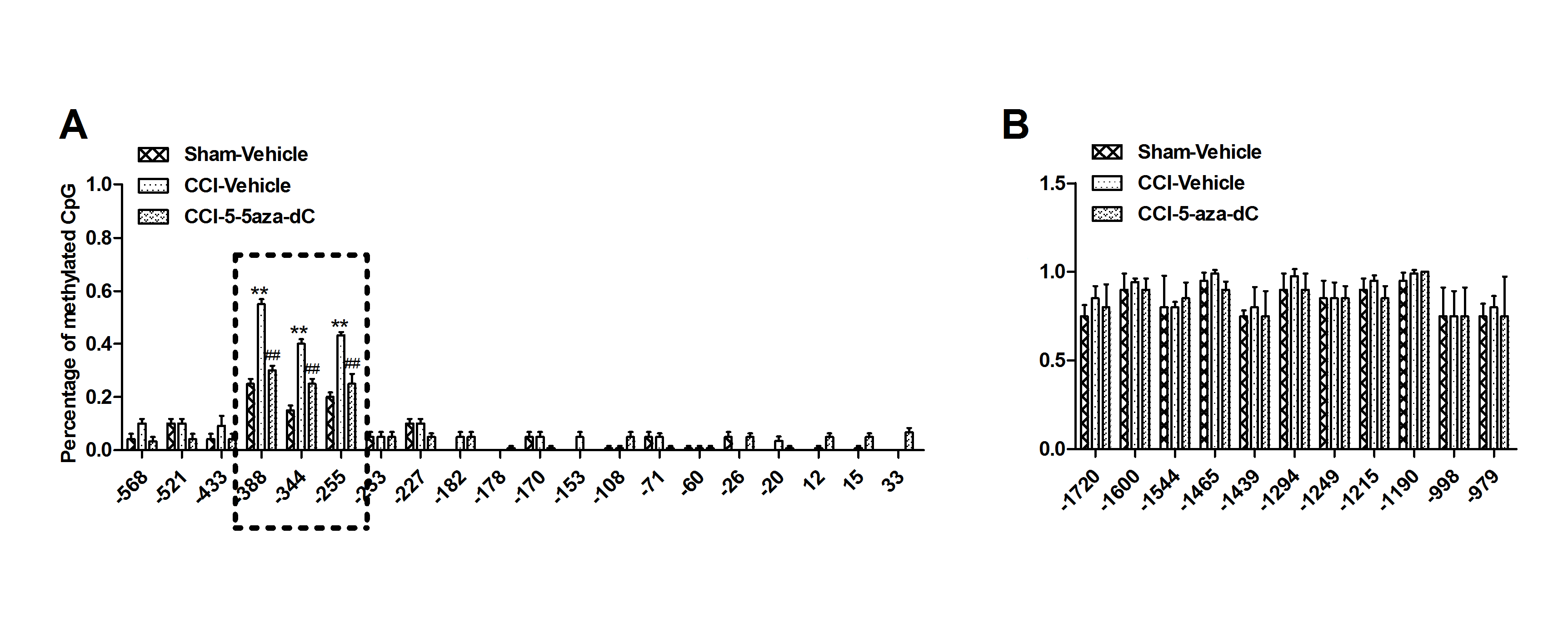

Supplement: Additional file 3: Figure S3 — A Methylation statuses of MOR gene PP in DRG. The percentages of methylation at CpG sites in the MOR promoter from the region of base pairs -569 to +33. -388, -344 and -255 CpG sites significantly increased in DRG after nerve injury and 5-aza-dC reduced the hypermethylation status. **P<0.01 compared with Sham-Vehicle mice, ##P<0.01compared with CCI-Vehicle mice, n=4 in each group. B, Methylation statuses of MOR gene DP in DRG. The percentages of methylation at CpG sites in the MOR promoter from the region of base pairs -1721 to -780. No CpG site has a significant change. n=4 mice in each group. [file 1744-8069-10-51-S3.tiff]

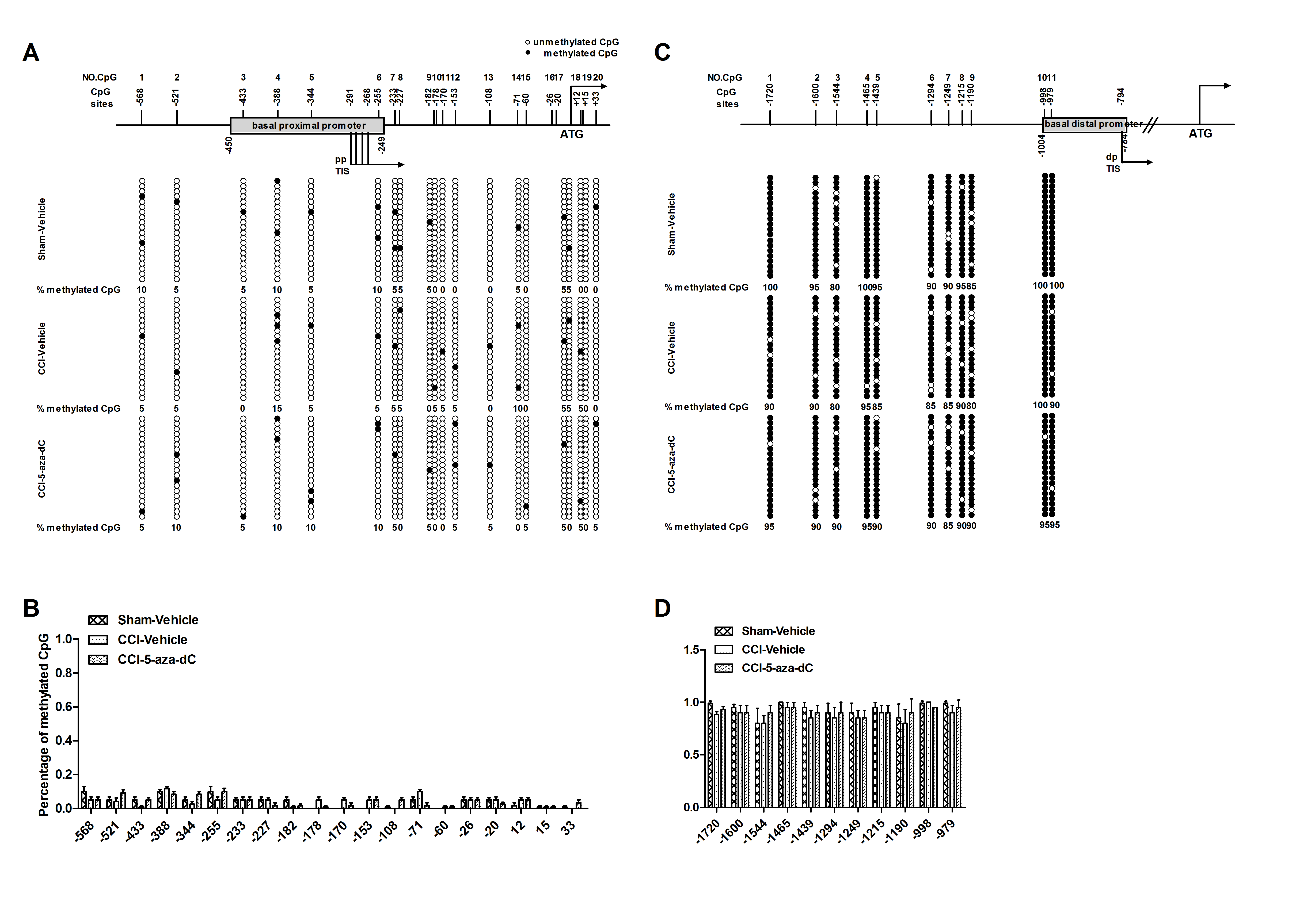

Supplement: Additional file 4: Figure S4 — A and B, methylation changes within the PP region of spinal MOR gene. No CpG site has a significant change. n=4 in each group. H, methylation changes within the DP region of spinal MOR gene. No CpG site has a significant change. n=4 in each group. [file 1744-8069-10-51-S4.tiff]
